# Effects of Omalizumab on Serum Levels of Substance P, Calcitonin Gene-Related Peptide, Neuropeptide Y, and Interleukin-31 in Patients with Chronic Spontaneous Urticaria

**DOI:** 10.1155/2023/8087274

**Published:** 2023-09-26

**Authors:** Cagdas Boyvadoglu, Hasan Ulusal, Seyithan Taysı, Goknur Ozaydin-Yavuz, Ibrahim Halil Yavuz, Pınar Korkmaz, Huseyin Serhat Inaloz

**Affiliations:** ^1^Department of Dermatology, Ceyhan State Hospital, Adana, Turkey; ^2^Department of Medical Biochemistry, University of Gaziantep Faculty of Medicine, Gaziantep, Turkey; ^3^Department of Dermatology, Yuzuncu Yil University Faculty of Medicine, Van, Turkey; ^4^Department of Dermatology, Ersin Arslan Training and Research Hospital, Gaziantep, Turkey; ^5^Department of Dermatology, University of Gaziantep Faculty of Medicine, Gaziantep, Turkey

## Abstract

The mechanism of action of omalizumab in urticaria is still not literally known. This study examines the serum values of substance P (SP), calcitonin gene-related peptide (CGRP), neuropeptide Y (NPY), and interleukin-31 (IL-31) in patients using omalizumab. In this study, 30 patients with chronic spontaneous urticaria (CSU) who were going to be treated with omalizumab and 20 healthy volunteers took part. Demographic data, clinical data, and disease activity scores were noted. For serum SP, CGRP, NPY, and IL-31 values, 10 mL of blood were taken from the patients before starting the treatment, 3 months after the treatment, at the end of the 6th month, and from healthy volunteers all at once. The change in values measured at baseline, 3rd month, and 6th month was analyzed by the Friedman Test. The Mann–Whitney *U* test was used to compare the parameters obtained from the patients and control groups. The significance level was set at *p*=0.05. SP, CGRP, NPY, and IL-31 values were all statistically significantly lower in the CSU patient group compared to the control group. After treatment, the levels of SP and CGRP in the serum went up, and the levels of serum IL-31 went down. These changes were statistically significant. This study supports the view that omalizumab does not only affect IgE receptors but also affects mast cells through other mechanisms. According to our knowledge, this is the first study to show that omalizumab therapy and serum CGRP levels are related.

## 1. Introduction

Omalizumab is an effective and safe treatment agent used in the treatment of chronic spontaneous urticaria (CSU) that is resistant to antihistamine therapy. It is a recombinant humanized immunoglobulin G1 monoclonal antibody that selectively binds to immunoglobulin E (IgE). It causes a decrease in circulating free IgE levels and downregulation of Fc epsilon receptors on the cell surface. Therefore, many mediators that are released from mast cells and cause urticaria are suppressed [1].

Neuropeptides such as substance P (SP), calcitonin gene-related peptide (CGRP), and neuropeptide Y (NPY) are released from free nerve endings with various chemical and physical stimuli. It is known that there is an interaction between mast cells and free nerve endings [2]. Therefore, neuropeptides are thought to be associated with mast cell activation and degranulation. SP is an important neuropeptide known to play a role in many pathophysiological pathways. It has also been shown to be associated with mast cell degranulation [3]. Previous studies have shown that there may be a relationship between SP and the development of chronic urticaria [4].

Another neuropeptide is CGRP, and it stands out with its powerful vasodilator feature. It was observed that, when administered intradermally, it statistically significantly increased the wheal and flare responses in CSU patients [5]. As far as we know, there is no study examining the effect of omalizumab treatment on serum CGRP levels.

NPY is a neuropeptide that is known to play an important role in pruritus but has not been previously associated with urticaria. It is known that it is released from neurons in response to stimuli that cause stress. It has been shown that there is a negative correlation between itching and serum NPY levels [6].

Interleukin-31 (IL-31) is a cytokine that is part of the IL-6 family. IL-31 is mainly secreted by T cells, but it is also known to be secreted by mast cells [7]. It has been suggested to be a major dermal pruritogen because it has been shown to play a very important role in itching in patients with atopic dermatitis [8]. It is well-known how it causes atopic dermatitis and pruritus, but it is still not clear what role it plays in urticaria.

Omalizumab has been used for many years, but its mechanism of action is still unknown. There is still no routinely recommended blood parameter to predict follow-up and response to treatment. It may also affect receptors on the mast cell surface in ways other than Fc epsilon RI.

In this study, the serum values of SP, CGRP, NPY, and IL-31 after treatment in CSU patients using omalizumab were examined.

## 2. Materials and Methods

Before starting the study, local ethics committee approval was obtained, and the study was initiated in accordance with the World Helsinki Declaration. Thirty CSU patients and 20 healthy volunteers participated in the study. Informed consent forms were obtained from patients and healthy volunteers participating in the study. The demographic characteristics of patients and healthy volunteers were recorded. The clinical features of the patients, such as the duration of the disease and the presence of angioedema, were also recorded. The Urticaria Control Test (UCT) and Urticaria Activity Score 7 (UAS7) were calculated and noted in the controls of the patients before and during the omalizumab treatment.

Blood was taken from the patient group at the beginning of the treatment, at the 3rd and 6th months of the follow-up, to measure the serum SP, CGRP, NPY, and IL-31 values. In order to measure the serum values of the same parameters, 10 mL of blood was taken at once from healthy volunteers. These blood samples were centrifuged for 5 min and then stored at −80° until measurement. Human ELISA Kit (Bioassay Technology Laboratory, China) was used to measure serum SP (E1528Hu), CGRP (E1061Hu), NPY (E1285Hu), and IL-31 (E3254Hu) values. Serum levels were analyzed according to the manufacturer's recommendations. It was read at a wavelength of 450 nm with automatic readers. Results are measured in nanograms/liter.

A total of 30 CSU patients were included in the study, six of whom were male and 24 were female. A total of 20 people, 10 women and 10 men, were included in the study as a healthy control group. While the mean age was 39.83 ± 11.67 years in the patient group, it was 31.85 ± 6.77 years in the healthy control group. It has been statistically shown that there is no relationship between serum SP, CGRP, NPY, and IL-31 levels and age. There were comorbidities in eight patients in the CSU patient group, while all of the volunteers in the control group were healthy. The mean disease duration of CSU patients was noted as 59.97 ± 77.51 months. In the CSU patient group, 10 patients had angioedema in addition to urticaria. Demographic and clinical data are summarized in Table 1.

### 2.1. Statistical Analysis

All statistical analyses were calculated by SPSS 22.0. Descriptive statistics of the data obtained from the study are given with mean and standard deviation for numerical variables, frequency, and percentage analysis for categorical variables. The conformity of the obtained biochemical parameters to the normal distribution was examined with the Shapiro–Wilk test. The change in values measured at baseline, 3rd month, and 6th month was analyzed by the Friedman test. If a significant difference was observed, we performed further post hoc analysis by using Dunn's test. The Mann–Whitney *U* test was used to compare the biochemical parameters obtained at the beginning of the study according to the patient and control groups. In addition, *t*-test and Chi-square tests were used to compare the demographic characteristics of the study groups. A significance level of *p* < 0.05 was chosen.

## 3. Results

UAS7 and UCT scores, calculated at the beginning of treatment and at follow-up, were noted (Table 2). A statistically significant decrease was observed in the UAS7 score at the 3rd and 6th month follow-ups compared to the beginning of the treatment. A statistically significant increase was observed in the UCT score at the 3rd and 6th month follow-ups compared to the beginning of the treatment. In our study, no statistically significant correlation was observed between serum SP, CGRP, NPY, and IL-31 levels and disease severity scores by the Spearman test.

The comparison of SP, CGRP, NPY, and IL-31 values in the patient and control groups is shown in Table 3. SP, CGRP, NPY, and IL-31 values were all statistically significantly lower in the CSU patient group compared to the control group.

**Figure 1:**
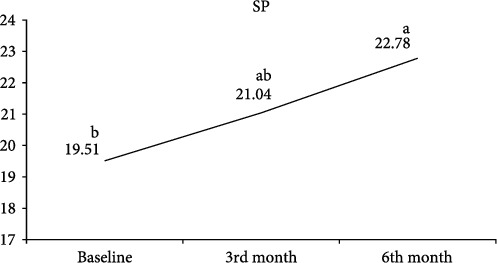
Graph of change in substance P. a, b: Different letters indicate which group the difference originates from (Dunn's test).

**Figure 2:**
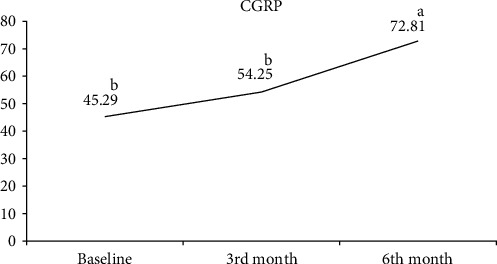
Graph of change in calcitonin gene-related peptide. a, b: Different letters indicate which group the difference originates from (Dunn's test).

**Figure 3:**
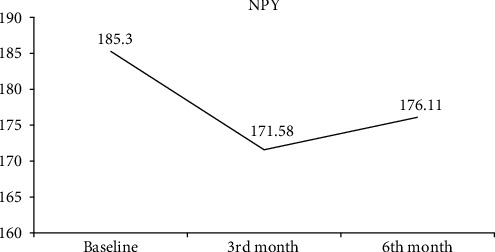
Graph of change in neuropeptide Y.

**Figure 4:**
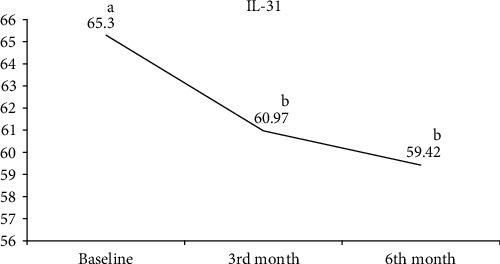
Graph of change in interleukin-31. a, b: Different letters indicate which group the difference originates from (Dunn's test).

The comparison of serum SP, CGRP, NPY, and IL-31 values measured at the beginning of treatment and at follow-ups in the CSU patient group is shown in Table 4. Serum SP (Figure 1) and CGRP (Figure 2) values increased continuously in the 3rd and 6th months of the treatment. A statistically significant increase in both parameters was observed in the 6th month of treatment. There was no statistically significant change observed in the serum NPY value before and after treatment (Figure 3). A statistically significant decrease was observed in the serum IL-31 value at the 3rd month of treatment. At 6 months of treatment, serum IL-31 levels were still statistically significantly lower than at the start of treatment (Figure 4).

## 4. Discussion

In our study, it was observed that serum SP, CGRP, NPY, and IL-31 values were lower in the CSU patient group than in the healthy control group. In addition, in our study, it was observed that serum SP and CGRP values increased, serum IL-31 values decreased, and serum NPY values did not change in CSU patients after omalizumab treatment. At the same time, after omalizumab treatment, a significant decrease in UAS7 scores and a significant increase in UCT scores were also observed.

The relationship between SP and urticaria has not been fully revealed, but many studies have shown that SP has a place in the pathogenesis of urticaria. SP is known to recognize some receptors on the surface of mast cells. It is a neuropeptide that has been shown to induce degranulation of mast cells in vitro [9]. It is known that SP can also cause mast cell degranulation by nonimmunological means. In our study, we found that the serum SP levels of the CSU patients were lower than those of the healthy volunteers. In contrast to our study, Metz et al. [4] have observed that serum SP values were higher in the CSU patient group compared to healthy volunteers. Mas-related G protein-coupled receptor X2 (MRGPRX2) has been suggested to be the main receptor on mast cells that are affected by neuropeptides. This receptor has been shown to play an important role in the regulation of allergic and inflammatory responses [10]. It has been shown that high levels of MRGPRX2 in CSU patients are associated with the severity of the disease. This finding suggests that MRGPRX2 has a potential role in the pathophysiology of the disease [11]. Activation of MRGPRX2 has been shown to increase skin reactivity in CSU patients. Activation of MRGPRX2 has been shown to degranulate mast cells and cause the release of proinflammatory mediators. It has been suggested that this may lead to increased inflammatory reactions in the skin [12]. Unfortunately, we could not check the level of MRGPRX2 in our study.

In a previous study, serum SP values of CSU patients who were given omalizumab were measured before and after treatment. Serum SP values went up in the 3rd and 6th months of the treatment, which is what has been seen [13]. In the same way, when omalizumab was used in our study, the serum SP values went up. A significant improvement was also observed in disease activity scores.

The role of CGRP in the pathogenesis of urticaria is another discussed neuropeptide. It is known to be involved in the development of vasodilation and inflammation. The CGRP-expressing cells of biopsy specimens taken from the lesioned skin of CSU patients and those of healthy volunteers were compared. CGRP-expressing cells were observed to be higher in the CSU patient group [14]. It has been observed that when CGRP was injected intradermally, the wheal and flare reaction was longer and worse in CSU patients than in healthy volunteers [6]. In the study of Başak et al. [15], it was observed that there was an increase in serum CGRP levels after treatment with H1 antihistamines in CSU patients. In our study, an increase in serum CGRP levels was found after treatment. Our study is the first to show the correlation between omalizumab treatment and serum CGRP values.

NPY is a neuropeptide that has been shown to be negatively correlated with itching. It has been observed in animal experiments that it suppresses itching [7]. In one study, the serum NPY values of psoriasis patients with and without pruritus were compared. It was observed that serum NPY levels were lower in psoriasis patients with pruritus [16]. In another study, it was observed that there was a decrease in serum NPY levels after treatment with H1 antihistamines in CSU patients [15]. To the best of our knowledge, no study has been reported to examine the relationship between serum NPY level and CSU. In our study, serum NPY values were found to be lower in CSU patients compared to the control group. However, there was no significant change in serum NPY values after omalizumab treatment.

IL-31 is known to be important in causing chronic inflammation of the skin. It has been shown to be linked to atopy, and its role in the development of atopic dermatitis has made it clear that it is the major dermal pruritogen [17, 18]. It has been shown to cause chemotaxis in basophils. This means that it might help with the transformation and migration of inflammatory cells in diseases like urticaria that cause inflammation [19]. In a study by Raap et al. [20], serum IL-31 levels in CSU patients were found to be significantly higher than in healthy controls, although not as high as in atopic dermatitis patients. In our study, serum IL-31 levels were significantly higher in the healthy control group. This finding is different from other studies in the literature. More studies are needed to obtain more information about the role of IL-31 in the pathogenesis of CSU.

In a previous study, serum IL-31 values were measured in CSU patients before and after 6 months of omalizumab treatment. It was observed that serum IL-31 levels decreased after omalizumab treatment. However, no correlation was observed between the severity of urticaria and IL-31 levels [21]. In another study, serum IL-31 values were measured in CSU patients before omalizumab treatment and at the 5th month of treatment. Serum IL-31 values were observed to decrease after omalizumab treatment [22]. In our study, a significant decrease in serum IL-31 values was observed after omalizumab treatment in CSU patients. This result may be due to the effect of omalizumab on the immunological response by causing changes in T cells, but it may also be the result of the downregulation of omalizumab in mast cells, which produces IL-31. Studies with a small number of patients in a single center confirm each other, but more studies involving large patient groups are needed to clearly reveal the relationship between them.

The limitations of our study are the demographic differences between the patient and control groups and the small number of patients who participated in the study. Our study could have been planned for a longer period. With a follow-up of 1 year or more, the results could have been different. Longer follow-up studies with larger and more heterogeneous patient groups are needed. In addition, we could not examine the level of MRGPRX2 in CSU patients in our study. To learn more about the pathogenesis of CSU, studies to examine the relationship between neuropeptides and MRGPRX2 level can be planned.

## 5. Conclusion

In this study, changes in serum SP, CGRP, NPY, and IL-31 values were observed during the 3rd and 6th months of omalizumab treatment. It is thought that omalizumab may also affect mast cells via immunological mechanisms through IgE-independent pathways. Our study resulted in supports for this view. However, it is still not clear how omalizumab works to treat urticaria, and more research is needed to learn more about it.

## Figures and Tables

**Table 1:** Demographical and clinical data: chronic spontaneous urticaria patient group and control group.

		CSU^†^ patient group	Control group	*p* Value
Age (average ± s.s.)		39.83 ± 11.67	31.85 ± 6.77	0.008^*∗*^
Disease duration (months) (average ± s.s.) (median (min–max))	
		59.97 ± 77.51 18.5 (3–252)		

Gender (*n*(%))	Woman	24 (80)	10 (50)	0.026^*∗*^
	Man	6 (20)	10 (50)	

Comorbidity (*n*(%))	(+)	8 (26.67)	0 (0)	0.012^*∗*^
	(−)	22 (73.33)	20 (100)	

Angioedema (*n*(%))	(+)	19 (63.33)		
	(−)	11 (36.67)		

*Note: t*-test and Chi-square tests. ^†^Chronic spontaneous urticaria.

**Table 2:** Change of urticaria control test and urticaria activity score 7.

	Baseline	3rd month	6th month	*p* Value
	Median (Q1–Q3)	Median (Q1–Q3)	Median (Q1–Q3)	
UAS7^†^	24.5 (16–42)^a^	2 (0–6)^b^	1.5 (0–8)^b^	0.001^*∗*^
UCT^‡^	3.50 (0–7)^b^	14.50 (11–16)^a^	15 (12–16)^a^	0.001^*∗*^

*Note:* Friedman test. ^†^Urticaria activity score 7, ^‡^Urticaria control test, ^a,b^Different letters indicate which group the difference originates from (Dunn's test).

**Table 3:** The comparison of serum substance P, calcitonin gene-related peptide, neuropeptide Y, and interleukin-31 values in the chronic spontaneous urticaria patient group and control group.

	CSU patient group	Control group	*p* Value
	Median (Q1–Q3)	Median (Q1–Q3)	
SP^†^	19.51 (14.51–26.89)	91.98 (60.05–114.19)	0.001^*∗*^
CGRP^‡^	45.29 (31.99–73.08)	348.54 (250.19–415.18)	0.001^*∗*^
NPY^§^	185.3 (135.06–270.26)	675.57 (423.17–793.78)	0.001^*∗*^
IL-31^¶^	65.30 (46.39–89.14)	169.57 (115.8–237.27)	0.001^*∗*^

*Note:* The Mann–Whitney *U* test. ^†^Substance P, ^‡^Calcitonin gene-related peptide, ^§^Neuropeptide Y, ^¶^Interleukin-31.

**Table 4:** Change in serum substance P, calcitonin gene-related peptide, neuropeptide Y, and interleukin-31.

	Baseline	3rd month	6th month	*p* Value
	Median (Q1–Q3)	Median (Q1–Q3)	Median (Q1–Q3)	
SP^†^	19.51 (14.51–26.89)^b^	21.04 (17.24–26.86)^ab^	22.78 (17.95–37.35)^a^	0.001^*∗*^
CGRP^‡^	45.29 (31.99–73.08)^b^	54.25 (41.46–74.84)^b^	72.81 (45.54–104.37)^a^	0.001^*∗*^
NPY^§^	185.3 (135.06–270.26)	171.58 (142.94–245.89)	176.11 (135.55–241.35)	0.872
IL-31^¶^	65.3 (46.39–89.14)^a^	60.97 (36.51–80.13)^b^	59.42 (39.61–78.9)^b^	0.020^*∗*^

*Note:* Friedman test.^†^Substance P, ^‡^Calcitonin gene-related peptide, ^§^Neuropeptide Y, ^¶^Interleukin-31. ^a,b^Different letters indicate which group the difference originates from (Dunn's test).

## Data Availability

All data are available from the corresponding authors under reasonable conditions.
